# Medicines for Obesity: Appraisal of Clinical Studies with Grading of Recommendations, Assessment, Development, and Evaluation Tool

**DOI:** 10.3390/nu15030606

**Published:** 2023-01-24

**Authors:** Eleni A. Karavia, Panagiota C. Giannopoulou, Vassiliki Konstantinopoulou, Katerina Athanasopoulou, Theodosios D. Filippatos, Demosthenes Panagiotakos, Kyriakos E. Kypreos

**Affiliations:** 1 Pharmacology Laboratory, Department of Medicine, University of Patras School of Health Sciences, Patras 26504, Greece; 2 Department of Internal Medicine, School of Medicine, University of Crete, Heraklion 71500, Greece; 3 School of Health Sciences and Education, Harokopio University, Athens 17676, Greece; 4 Department of Life Sciences, School of Sciences, European University Cyprus, Nicosia 1516, Cyprus

**Keywords:** obesity, GRADE evaluation, clinical pharmacology, clinical evidence

## Abstract

We evaluated the quality of evidence from phase III/IV clinical trials of drugs against obesity using the principles of Grading of Recommendations, Assessment, Development, and Evaluation (GRADE) tool. Our systematic review evaluates the quality of clinical evidence from existing clinical trials and not the pharmacological efficacy of anti-obesity therapies. A literature search using select keywords in separate was performed in PubMed and ClinicalTrials.gov databases for phase III/IV clinical trials during the last ten years. Our findings indicate that the quality of existing clinical evidence from anti-obesity trials generally ranges from low to moderate. Most trials suffered from publication bias. Less frequently, trials suffered from the risk of bias mainly due to lack of blindness in the treatment. Our work indicates that additional higher-quality clinical trials are needed to gain more confidence in the estimate of the effect of currently used anti-obesity medicines, to allow more informed clinical decisions, thus reducing the risk of implementing potentially ineffective or even harmful therapeutic strategies.

## 1. Introduction

“Lifestyle diseases” is a contemporary, modern definition used to describe a range of disorders related to unhealthy habits such as excess food consumption and caloric intake, low physical activity, and disturbed circadian clock. They include coronary heart and fatty liver disease, obesity, and type 2 diabetes mellitus (T2DM) [1]. These disorders affect a very large part of the global population, and even though current treatment strategies are constantly updated, including the use of novel drugs, there are still numerous unmet medical needs to be addressed and many pharmacological challenges to tackle.

Obesity is a chronic, metabolic disorder characterized by increased fat depots, either in number or in size [2,3]. It is considered as the central pathology of metabolic syndrome, which may also include two of the following conditions: hypertension, disturbed plasma glucose homeostasis, and a distinct type of dyslipidemia characterized by increased plasma triglyceride levels and reduced HDL cholesterol levels. Phenotypically, obesity combines increased body mass, mainly due to adipose tissue expansion, and lean mass augmentation [4]. Undoubtedly, a prolonged positive energy balance contributes to the development of the disease [5]; yet, many other biological, environmental, and social parameters may also be implicated in the etiology of the disease [5,6]. In 2016, the World Health Organization reported that 39% of adults were overweight and 13% were obese, while the prevalence of overweight and obesity among children and adolescents was 18%. Impressively, it was reported that in 2019, 38 million children younger than the age of five were overweight and obese [7] (accessed on 24 June 2022). Obesity, abdominal fat, and dysfunctional adipose tissue are associated with the development of cardiovascular diseases, T2DM, several cancers, and hepatic or renal diseases [8], which are considered some of the primary causes of disability and death [9]. Therapeutic lifestyle changes (TLCs), such as changes in dietary habits and exercise, are often associated with significant weight loss and a normalization of body weight. Nevertheless, often times TLCs fail to produce sustained results and the use of medicines is mandated. In cases of morbid obesity with very high body mass indices, surgical weight loss appears the most effective solution. In recent years, bariatric surgical interventions have been used very effectively for sustained weight loss and normalization of plasma lipid levels, glucose tolerance, and insulin sensitivity in morbidly obese patients [10]. In particular, malabsorptive bariatric procedures have been proven more effective compared to restrictive bariatric procedures aiming at reducing food intake [11].

Therapeutic strategies for obesity aim not only at weight loss but also at prevention of weight regain [6,12], refinement of body composition, treatment of comorbidities, decrease in health risk, and improvement of life quality. Both lifestyle interventions and pharmacotherapy contribute significantly to this direction [6,12,13]; however, the use of medicines is usually recommended only for individuals with a body mass index (BMI) ≥30 kg/m^2^ without comorbidities or a BMI ≥27 kg/m^2^ if at least one obesity-related comorbidity, such as dyslipidemia or diabetes, is present [14]. Currently, both the Food and Drug Administration (FDA) and the European Medicines Agency (EMA) have given their approval to semaglutide orlistat, liraglutide, and the combination of naltrexone/bupropion for the indication of obesity. The combination of phentermine/topiramate is approved only by the FDA. These active pharmaceutical compounds (APC) act through various mechanisms; for example, orlistat diminishes the intestinal digestion of lipids via highly specific inhibition of pancreatic and gastric lipases in the gastrointestinal tract [15], while liraglutide and semaglutide are long-acting agonists for the receptor of glucagon-like peptide-1 (GLP-1) [16]. Notably, the approved fixed-dose combinations aim at improving effectiveness and safety [12]. Particularly, a pooled-data clinical trial showed that phentermine/topiramate combination therapy is more effective in reducing body weight compared to either topiramate or phentermine monotherapies [17]. The combination of an opioid receptor antagonist (naltrexone) with a dopamine and norepinephrine reuptake inhibitor (bupropion) in an extended-release tablet is more efficacious in terms of weight loss compared with bupropion monotherapy [18].

The use of medicines for obesity poses a significant financial burden on health care systems all over the world and it should be mandated by strong clinical evidence supporting their efficacy. In the absence of quality clinical data, the risk and cost may far exceed the benefit, advocating against the use of these drugs.

In the present systematic review, we applied the Grading of Recommendations, Assessment, Development, and Evaluation (GRADE) tool [19] to assess if medicines currently used for obesity are effective based on the quality of existing clinical evidence. Addressing this question could also be very important for health technology assessment organizations and guideline committees, in assisting their decisions.

## 2. Methods

GRADE is typically an instrument to evaluate a body of evidence for a particular patient/intervention/comparison/outcome, also known as PICO. Briefly, GRADE provides a methodology for the classification of quality of evidence focusing on specific quality factors [20]. These factors include risk of bias [21], inconsistency [22], indirectness [23], imprecision [24], and publication bias [25], all affected by individual parameters, as presented in brief in Table 1. In our paper, emphasis is placed on the evaluation of clinical evidence rather than on the efficacy of each medicine, which is not a subject of GRADE; that is, to determine what the evidence from a set of studies for each drug tells us about a given PICO. To facilitate our work, prior to GRADE implementation in each set of trials, every single trial was pre-evaluated based on critical “YES or NO” questions regarding the GRADE factors shown in Table 1. The limiting factors, which, according to GRADE, were identified to affect the quality of evidence in each trial are listed in Table 2 and in more detail, in Table S1 of Supplementary Data.

Following this initial screening of individual trials, for each drug, we then proceeded to an overall evaluation of the body of relevant clinical evidence from all studies, according to the GRADE handbook: https://gdt.gradepro.org/app/handbook/handbook.html [49] (accessed on 24 June 2022), and the results are listed in Table 3. The evaluation of clinical evidence for each PICO was performed using the academic version of *GRADEpro* tool (GRADEpro GDT: GRADEpro Guideline Development Tool [Software], McMaster University, 2020 (developed by Evidence Prime, Inc., available from gradepro.org, accessed on 10 January 2023)). The results of the overall evaluations for the anti-obesity drugs are presented in Supplemental Data.

The clinical trials included in the present study were extracted from PubMed using the following keywords in separate: “obesity”, “anti-obesity drugs”, “anti-obesity effects”, “anti-obesity drug effectiveness”, “anti-obesity medicine effectiveness”, “orlistat obesity effectiveness”, “liraglutide obesity effectiveness”, “naltrexone/bupropion obesity effectiveness”, “semaglutide obesity effectiveness” “phentermine obesity effectiveness”, “topiramate obesity effectiveness”, and the following filters were applied: Clinical Trial, Phase III, Clinical Trial, Phase IV, 10 years, Humans, English. The search retrieved studies published over the last 10 years, based on human species and in English language. In addition, the ClinicalTrials.gov database was used to identify additional studies and collect more relevant information. Advanced research was performed with the following fields: “Condition or disease: obesity”, “Other terms: weight loss or weight loss effect or orlistat or liraglutide or naltrexon/bupropion or semaglutide”, “Study Types: All Studies”, “Study Results: Studies with results”, “Phase: 3”, “Phase: 4”, “Study start: From 1 January 2012to 8 November 2022”. The following characteristics were extracted from the original papers using a standardized data extraction form: design of the study, lead author, year of publication, country of origin, and sample size. The drugs evaluated in this systematic review are drugs tested in multiple clinical trials, namely, naltrexone/bupropion, liraglutide, lorcaserin, semaglutide, and exenatide. Other drugs, such as bupropion, phentermine/topiramate, empagliflozin, ertugliflozin, amfepramone, rimonabant, setmelanotide, glargine, and tirzepatide, found only in single clinical trials were not included since GRADE is not applied to single studies.

**Figure 1:**
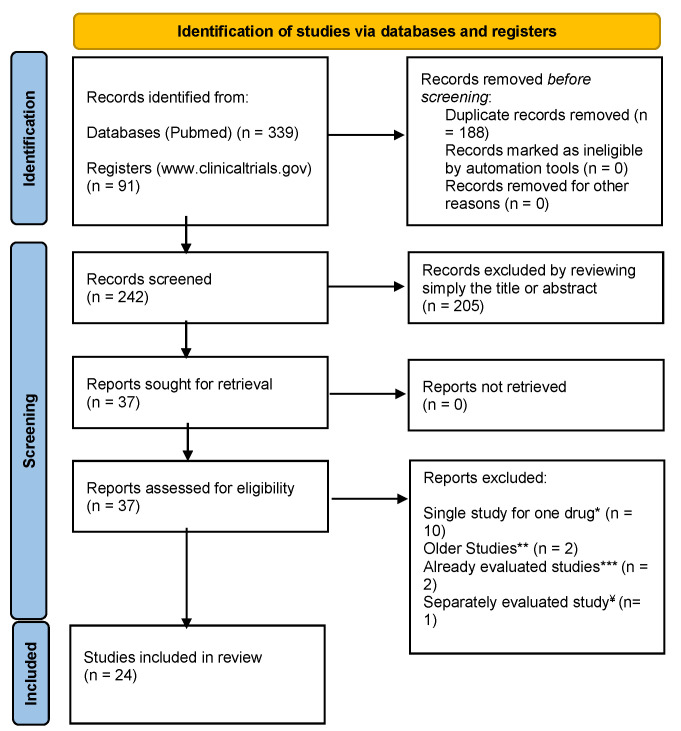
Strategy for collecting and evaluating clinical evidence for anti-obesity medicines in the context of this systematic review. PRISMA 2020 flow diagram for new systematic reviews, which included searches of databases and registers only. * GRADE cannot be applied in a single study; thus, in this review, drugs found in only one study were excluded. ** Studies that include clinical trials from 2009 were excluded since our research focuses on the last ten years. *** Studies that include clinical trials evaluated in other included studies. ¥ This study includes two different studies, which we evaluated separately; thus, the number of “Studies included in review” is 24 and not 22 [51].

PCG, VK, and KA performed a literature search independently, and EAK, GP, and KEK participated in the independent appraisal of the collected manuscripts and GRADE evaluation of the clinical evidence. Disagreements were solved by consensus and by the expert opinion of TF, AK, and DP. All authors contributed to the drafting of the final submitted version of the manuscript. The search and sorting strategies for our qualitative synthesis are summarized in Figure 1. The PRISMA flow diagram was created according to the guidelines described in [50].

## 3. Results

### 3.1. Naltrexone/Bupropion

The combination of naltrexone, an opioid μ-receptor antagonist, with bupropion, a dual norepinephrine and dopamine reuptake inhibitor, was approved by the FDA for obesity treatment in 2014.

A clinical trial (CONTRAVE Obesity Research-II (COR-II), NCT00567255) [26] showed that 1496 individuals with obesity (BMI: 30–45 kg/m^2^) or overweight (27–45 kg/m^2^) and with dyslipidemia and/or hypertension who received naltrexone/bupropion experienced greater weight loss compared to the placebo group.

Another clinical trial (NCT01764386) [27] showed that in 242 subjects with obesity or overweight with dyslipidemia and/or controlled hypertension, treatment with naltrexone SR/bupropion SR in conjunction with a lifestyle intervention program for 26 weeks improved weight loss, which was sustained for 78 weeks, compared to usual care.

The clinical evidence supporting the effectiveness of naltrexone/bupropion combination compared to placebo or usual care for weight loss is of low quality (Table 2 and Table 3).

### 3.2. Liraglutide

Liraglutide is a GLP-1 agonist used for the improvement of glycemic control in patients with type 2 diabetes mellitus (T2DM). Different clinical trials indicated a beneficial effect of GLP-1 analogs on obesity; thus, liraglutide was also licensed as a weight loss agent, with encouraging results in phase III clinical trials [34,52].

A recent post hoc analysis of pooled data from four phase IIIa trials in patients with either a minimum BMI of 27 kg/m^2^ and (at least) one comorbidity or a minimum BMI of 30 kg/m^2^ compared the efficacy and safety of liraglutide versus placebo, combined with a low-calorie diet and physical activity (NCT01272219, NCT01272232, NCT01557166, NCT00781937) [28]. The study showed that both Hispanic (*n* = 534) and non-Hispanic (*n* = 4597) subgroups had clinically significant mean weight loss after treatment with liraglutide, while more patients in both subgroups lost ≥5%, >10%, and >15% of their initial weight compared to placebo.

Furthermore, another randomized placebo-controlled, 12-week clinical trial in 72 overweight individuals and individuals with obesity, all of which had type 1 diabetes as comorbidity, showed that the addition of an either high (1.2 and 1.8 mg) or low (0.6 mg) liraglutide dose to insulin therapy resulted in significant weight reduction (NCT01722266) [29].

A 56-week, randomized, double-blind, placebo-controlled, multinational, multicenter trial with 396 participants (SCALE™ Insulin, NCT02963922) [30] showed that overweight individuals or individuals with obesity, all of which were insulin-treated for T2DM, increased weight loss compared to placebo when liraglutide was added as an adjunct to intensive behavioral therapy (IBT).

Similarly, a 56-week, randomized, double-blind, placebo-controlled, multicenter trial with 282 participants (SCALE™ IBT, NCT02963935) [31] showed that at week 56, the mean weight loss with liraglutide plus IBT was greater compared to placebo combined with IBT.

Furthermore, a randomized, double-blind trial (NCT02918279) showed that the use of liraglutide (3.0 mg) for 56 weeks plus lifestyle therapy led to a significantly greater reduction in the BMI standard deviation score compared to placebo plus lifestyle therapy in adolescents with obesity [32].

Moreover, in a 56-week, double-blind trial involving 3731 patients with either a minimum BMI of 30 kg/m^2^ or a minimum BMI of 27 kg/m^2^ with treated or untreated dyslipidemia or hypertension, treatment with 3.0 mg of liraglutide, as an adjunct to diet and exercise, was associated with reduced body weight and improved metabolic control (NCT01272219) [33].

Additionally, the use of subcutaneous liraglutide (3.0 mg) daily as an adjunct to 500 kcal/d dietary deficit and increased physical activity, compared to placebo, resulted in weight loss in overweight individuals and participants with obesity and T2DM over 56 weeks (NCT01272232) [34].

Finally, a clinical trial (NCT02911818) with 150 adults with obesity suggested a potential benefit on weight loss from adding 3.0 mg liraglutide to IBT for obesity compared to IBT alone [53].

Overall, the current clinical evidence for liraglutide is of moderate quality (Table 2 and Table 3).

### 3.3. Lorcaserin

Lorcaserin is a selective 5-HT_2C_ receptor agonist with indication for weight management, in combination with lifestyle modification, for overweight participants or participants affected by obesity with more than one weight-related comorbidity [54].

Two phase III clinical trials of similar design, the Behavioral Modification and Lorcaserin for Overweight and Obesity Management (BLOOM, NCT00395135, *n* = 3182) and the Behavioral Modification and Lorcaserin Second Study for Obesity Management (BLOSSOM, NCT00603902, *n* = 4008), assessed the safety and effectiveness of lorcaserin in adults without T2DM. A pooled analysis of these clinical trials showed that lorcaserin-treated patients, in combination with diet and physical activity, lost significantly more body weight compared to the placebo group [35].

Moreover, a post hoc analysis of the BLOOM-DM trial (NCT00603291) suggested that lorcaserin treatment of overweight patients and patients affected by obesity with T2DM for 52 weeks promoted weight reduction and facilitated glycemic control compared to placebo [36].

Finally, in a 12-week, randomized, double-blind pilot safety study (NCT01987427) with 238 nondiabetic patients with obesity or overweight with more than one comorbidity, the use of lorcaserin added to phentermine enhanced short-term weight loss [37].

Overall, current clinical evidence for lorcaserin is of moderate quality (Table 2 and Table 3). On 13 February 2020, FDA requested that the manufacturer of pharmaceutical products containing the active ingredient lorcaserin voluntarily withdraw the weight loss drug from the U.S. market because a safety clinical trial showed an increased occurrence of cancer [55] (accessed on 24 June 2022) .

### 3.4. Semaglutide

In 2014, the FDA approved a subcutaneous injection of semaglutide, a GLP-1 receptor agonist, (2.4 mg once weekly) for chronic weight management in adults with a BMI ≥30 kg/m^2^ or BMI ≥27 kg/m^2^ with at least one obesity-related disorder. The use of the drug is recommended along with a low-calorie diet and increased physical activity.

In a double-blind trial (NCT03548935) with 1961 adults with a BMI ≥30 kg/m^2^ or a BMI ≥27 kg/m^2^ with at least one weight-related coexisting condition, except T2DM, semaglutide was administered subcutaneous once weekly for 68 weeks at a dose of 2.4 mg in combination with lifestyle intervention. Compared to placebo, semaglutide was associated with a sustained, clinically relevant reduction in body weight [38].

A phase IIIa, open-label, parallel-group, randomized controlled trial (SUSTAIN 3, NCT01885208) [39] with 813 participants with T2DM indicated that semaglutide was superior to exenatide extended-release in improving glycemic control and reducing body weight at week 56 of treatment.

A randomized, double-blind study (STEP 3, NCT03611582) including 611 overweight adults or adults affected by obesity without diabetes showed that once-a-week subcutaneous semaglutide, used as an adjunct to IBT and an initial low-calorie diet, compared to placebo, resulted in significantly greater weight loss over 68 weeks [40].

Furthermore, treatment once a week of subcutaneous semaglutide at a does of 2.4 mg plus a lifestyle intervention for 68 weeks for weight management in overweight adults or adults with obesity and T2DM achieved a superior decrease in body weight compared to placebo (STEP 2, NCT03552757) [41].

Moreover, a randomized, open-label, 68-week trial showed that among 338 overweight patients or patients affected by obesity without diabetes, once-weekly subcutaneous semaglutide, compared to once-daily subcutaneous liraglutide in combination with counseling for diet and physical activity, resulted in significantly greater weight loss (STEP 8, NCT04074161) [42].

In addition, in a phase IIIb, open-label trial where 577 adults with T2DM were randomized to subcutaneous once-weekly semaglutide (1.0 mg) or subcutaneous once-daily liraglutide (1.2 mg), semaglutide was superior to liraglutide in reducing body weight (SUSTAIN 10, NCT03191396) [43].

Another randomized, double-blind, 68-week phase IIIa withdrawal study suggested that in patients who completed a 20-week run-in period with subcutaneous semaglutide, 2.4 mg once weekly, maintaining treatment with semaglutide compared to switching to placebo resulted in continued weight loss over the following 48 weeks (STEP 4, NCT03548987) [44].

Finally, another clinical study with adults with T2DM who were treated with a sodium glucose co-transporter-2 (SGLT-2) inhibitor and were randomly assigned to receive subcutaneous semaglutide (1.0 mg) or placebo once weekly for 30 weeks showed that adding semaglutide to SGLT-2 inhibitor therapy significantly reduce bodyweight (SUSTAIN 9, NCT03086330) [45].

Overall, current clinical evidence for semaglutide is of low quality (Table 2 and Table 3).

### 3.5. Exenatide

Exenatide, another GLP-1 receptor agonist [56], is indicated as an adjunct to diet and exercise to improve glycemic control in adults with T2DM; however, it, also appears to reduce body weight in clinical trials [57].

A recent randomized, multicenter, double-blind study (DURATION-7, NCT02229383) including 464 patients with persistent hyperglycemia showed that exenatide once weekly significantly decreased body weight, a secondary clinical outcome of the trial, compared to placebo [46].

Recently, exenatide was evaluated in combination with dapagliflozin, another antidiabetic drug that inhibits SGLT-2 in the kidney. Specifically, in a post hoc analysis of 695 patients with T2DM inadequately controlled with metformin monotherapy (subpopulations from the DURATION-8 trial, NCT02229396), it was found that 28-week treatment with exenatide once weekly plus dapagliflozin resulted in a body weight reduction that was no greater than that observed with exenatide once weekly or dapagliflozin alone [47]. For each group, weight loss was numerically greater as baseline BMI increased.

Finally, a randomized, placebo-controlled study including 182 overweight women and women with obesity (NCT01590433) showed that in women who demonstrated ≥5% weight loss at 12 weeks, longer-term weight loss with exenatide treatment was similar to that achieved with a hypocaloric diet [48].

The use of exenatide compared to placebo or dapagliflozin for weight loss is based on evidence of low quality (Table 2 and Table 3).

## 4. Conclusions

GRADE is composed of several criteria aimed at evaluating the quality of a body of evidence for a particular patient cohort, intervention, population, and outcome. Even though the assessment of some GRADE criteria may be subjected to the opinion of the evaluator (for example, the type of a study population or the magnitude of effect), most of them are largely objective in nature as they pertain to the design of the clinical trial. Indeed, GRADE has gained global acceptance and has been endorsed by a large number of societies and institutions in many different medical specialties [58,59,60].

Based on our assessment, the quality of evidence from most trials assessing anti-obesity medicines ranges from low to moderate (Table 3). As such, in cases where the rating was low, further research is very likely to have an important impact on our confidence in the estimate of effect and is likely to change the estimate. In all other cases where the rating of the quality of evidence was moderate, further research is likely to have an impact, and may change the estimate. Most trials suffer from publication bias since the evidence arose from small trials (number of participants < 1000) funded by the marketing authorization holder. Less frequently, trials suffered from the risk of bias mainly due to a lack of blindness in the allocation of treatment(s). In addition, imprecision due to inappropriate optimal information size was found in a few trials, while inconsistency was not found.

Though the quality of evidence is important, we should keep in mind that clinical recommendations may consider other parameters such as balance between desirable and undesirable effects, patient values, patient preferences, and treatment cost. Therefore, it should be noted that in addition to objective criteria for deciding on a rating, GRADE also includes some subjective parameters that may be hard to evaluate [61,62].

Our work is unique and quite novel in its nature, context, and impact in the sense that in addition to reviewing phase III/IV clinical evidence, it engages in a critical assessment of this evidence based on the strict methodology and impartial, highly objective approach of GRADE. Despite encouraging data, some crucial questions remain regarding the quality of clinical evidence that supports the effectiveness of many of these drugs. It should be noted that our systematic review evaluates the quality of clinical evidence from existing clinical trials and not the pharmacological efficacy of anti-obesity therapies. In addition, in our work, we did not perform a comparison of pharmacological efficacy among different medications. In the absence of head-to-head clinical trials, comparing different APCs for efficacy would lead to the activation of the indirectness criterion, which would further reduce the confidence in clinical evidence. A limitation that we identified in our study is the fact that our analysis did not include studies currently in progress whose data have not been published yet. Such studies, when completed, could impact the quality of clinical evidence. Overall, our findings raise the possibility that additional higher-quality, free-of-limitations clinical trials are needed to gain more confidence in the estimate of the effect of currently used anti-obesity medicines and to allow more informed clinical decisions, thus reducing the risk of implementing potentially ineffective or even harmful therapeutic strategies.

## Figures and Tables

**Table 1:** Critical questions to evaluate the clinical evidence of each study, based on GRADE factors.

QUESTIONS	ACTION
**1. Risk of Bias**	
1a. Randomized?	Yes = maintain, No = downgrade
1b. Double blind?	Yes = maintain, No = downgrade
1c. Dropouts/Withdrawals accounted for?	Yes = maintain, No = downgrade
1d. Large loses to follow-up?	Yes = downgrade, No = maintain
1e. All data taken into consideration?	Yes = maintain, No = downgrade
1f. Adherence to intention to treat analysis?	Yes = maintain, No = downgrade
1g. Stop early for benefit?	Yes = downgrade, No = maintain
1h. Failure to report outcomes?	Yes = downgrade, No = maintain
**2. Inconsistency**	
2a. Population Heterogeneity?	Yes = downgrade, No = maintain
2b. Intervention Heterogeneity?	Yes = downgrade, No = maintain
2c. Outcome heterogeneity?	Yes = downgrade, No = maintain
**3. Indirectness**	
3a. Indirect comparison?	Yes = downgrade, No = maintain
3b. Study population differs from target population?	Yes = downgrade, No = maintain
3c. Comparator differs among studies?	Yes = downgrade, No = maintain
3d. Study outcomes differ from outcomes of interest?	Yes = downgrade, No = maintain
**4. Imprecision**	
4a. Optimal information size (OIS)?	Yes = maintain, No = downgrade
4b. Mean difference 95% CI includes 0?	Yes = downgrade, No = maintain
4c. Mean 95% CI ranges overlap?	Yes = downgrade, No = maintain
4d. Risk ratio 95% CI includes 1?	Yes = downgrade, No = maintain
**5. Publication bias**	
5a. Failure to report studies, especially those showing no effect?	Yes = downgrade, No = maintain
5b. Evidence arises from small trials funded by the drug company?	Yes = downgrade, No = maintain

**Table 2:** Drugs, ATC, NCT, and the bias (limitations) found in each study. The factor contributing to bias according to Table 1 is indicated in parenthesis.

Name	Anatomical Therapeutic Chemical (ATC)	Authors/National Clinical Trial (NCT)	Bias (Type Based on Table 1)
Naltrexone/Bupropion	A08AA62	Apovian et al. [26]/NCT00567255	None
		Halseth et al. [27]/NCT01764386	Risk of bias (1a), Publication bias (5b)
Liraglutide	A10BJ02	O’Neil et al. [28]/NCT01272219, NCT01272232, NCT01557166, NCT00781937	None
		Kuhadiya et al. [29]/NCT01722266	Imprecision (4a)
		Garvey et al. [30]/NCT02963922	Publication bias (5b)
		Wadden et al. [31]/NCT02963935	Publication bias (5b)
		Kelly et al. [32]/NCT02918279	Publication bias (5b)
		Pi-Sunyer et al. [33]/NCT01272219	None
		Davies et al. [34]/NCT01272232	Publication bias (5b)
		Wadden et al./NCT02911818	Risk of bias (1b), Imprecision (4a), Publication bias (5b)
Lorcaserin	A08AA11	Aronne et al. [35]/NCT00395135, NCT00603902	Risk of bias (1d)
		Pi-Sunyer et al. [36]/NCT00603291	Publication bias (5b)
		Smith et al. [37]/NCT01987427	None
Semaglutide	A10BJ06	Wilding et al. [38]/NCT03548935	None
		Ahmann et al. [39]/NCT01885208	Risk of bias (1a), Publication bias (5b)
		Wadden et al. [40]/NCT03611582	Publication bias (5b)
		Davies et al. [41]/NCT03552757	None
		Rubino et al. [42]/NCT04074161	Risk of bias (1a), Inconsistency (2b), Publication bias (5b)
		Capehorn et al. [43]/NCT03191396	Risk of bias (1a), Publication bias
		Rubino et al. [44]/NCT03548987	Publication bias (5b)
		Zinman et al. [45]/ NCT03086330	Publication bias (5b)
Exenatide	A10BJ01	Guja et al. [46]/NCT02229383	Publication bias (5b)
		Jabbour et al. [47]/NCT02229396	Imprecision (4a), Publication bias (5b)
		Rodgers et al. [48]/NCT01590433	Imprecision (4a), Publication bias (5b)

**Table 3:** Assessment of quality using the academic version of GRADEpro tool.

	No. of Studies	Risk of Bias	Inconsistency	Indirectness	Imprecision	Risk of Publication Bias	Total Assessment
Naltrexone/ Bupropion	2	Serious	Not serious	Not serious	Not serious	Strongly suspected	Low
Liraglutide	8	Not serious	Not serious	Not serious	Not serious	Strongly suspected	Moderate
Lorcaserin	3	Not serious	Not serious	Not serious	Not serious	Strongly suspected	Moderate
Semaglutide	8	Serious	Not serious	Not serious	Not serious	Strongly suspected	Low
Exenatide	3	Not serious	Not serious	Not serious	Very serious	Strongly suspected	Low

## Data Availability

Not Applicable.

## Supplementary Materials

The following supporting information can be downloaded at https://www.mdpi.com/article/10.3390/nu15030606/s1, Table S1: Results from the PICO analysis for each medicine evaluated in the study. The factors identified to affect the quality of clinical evidence are listed in detail in the Explanations under each PICO table.
