# Supplementary material for: Medicines for Obesity: Appraisal of Clinical Studies with Grading of Recommendations, Assessment, Development, and Evaluation Tool
Source: Nutrients. 2023 Jan 24;15(3):606. doi: 10.3390/nu15030606 (PMC9919203; doi:10.3390/nu15030606)
Supplement: Supplementary file 1 [file nutrients-15-00606-s001.zip › nutrients-2137131-supplementary.pdf]

Medicines for Obesity: Appraisal of Clinical Studies with Grading of Recommendations, Assessment, Development, and Evaluation Tool

Eleni A. Karavia [1], Panagiota C. Giannopoulou [1], Vassiliki Konstantinopoulou [1], Katerina Athanasopoulou [1], Theodosios D. Filippatos [2], Demosthenes Panagiotakos [3] and Kyriakos E. Kypreos [1,4,*]

[1] Pharmacology Laboratory, Department of Medicine, University of Patras School of Health Sciences, 26504 Patras, Greece
[2] Department of Internal Medicine, School of Medicine, University of Crete, 71500 Heraklion, Greece
[3] School of Health Sciences and Education, Harokopio University, 17676 Athens, Greece
[4] Department of Life Sciences, School of Sciences, European University Cyprus, 1516 Nicosia, Cyprus
* Correspondence: kkypreos@upatras.gr; Tel.: +30-261-096-9120

Table S1: Results from the PICO analysis for each medicine evaluated in the study. The factors identified to affect the quality of clinical evidence are listed in detail in the Explanations under each PICO table.

Question: Naltrexone/Bupropion compared to placebo or usual care for weight loss

Certainty assessment							№ of patients		Effect		Certainty	Importance
№ of studies	Study design	Risk of bias	Inconsistency	Indirectness	Imprecision	Other considerations	Naltrexone/Bupropion	placebo or usual care	Relative (95% CI)	Absolute (95% CI)		
**weight loss**												
2	randomised trials	serious[a]	not serious	not serious	not serious	publication bias strongly suspected[b]					⊕⊕◯◯ Low	

CI: confidence interval

Explanations: a. The study by Halseth et al. (2017) is an open-label study, b. In the study by Halseth et al. (2017), evidence arises from a small trial with 300 patients funded by the drug company

Question: Liraglutide compared to placebo for weight loss

Certainty assessment							№ of patients		Effect		Certainty	Importance
№ of studies	Study design	Risk of bias	Inconsistency	Indirectness	Imprecision	Other considerations	Liraglutide	placebo	Relative (95% CI)	Absolute (95% CI)		
**weight loss**												

Certainty assessment							№ of patients		Effect		Certainty	Importance
№ of studies	Study design	Risk of bias	Inconsistency	Indirectness	Imprecision	Other considerations	Liraglutide	placebo	Relative (95% CI)	Absolute (95% CI)		
8	randomised trials	not serious	not serious	not serious	not serious	publication bias strongly suspected[a]					⊕⊕⊕◯ Moderate	

CI: confidence interval

Explanations: a. The studies by Garvey et al. (2020), Wadden et al. (2020), Kelly et al. (2020), Davies et al. (2015), and Wadden et al. (2019) are small trials with 396, 282, 251, 846 and, 150 participants, respectively, which are funded by the drug companies

Question: Lorcaserin compared to placebo for weight loss

Certainty assessment							№ of patients		Effect		Certainty	Importance
№ of studies	Study design	Risk of bias	Inconsistency	Indirectness	Imprecision	Other considerations	Lorcaserin	placebo	Relative (95% CI)	Absolute (95% CI)		
**weight loss**												
3	randomised trials	not serious[a]	not serious	not serious	not serious	publication bias strongly suspected[b]					⊕⊕⊕◯ Moderate	

CI: confidence interval

Explanations: a. Only 1 study (Aronne et al., 2014) out of 3, had a withdrawal rate of almost 50% at 1 year, b. The study by Pi-Sunyer et al. (2016) is a small trial with 604 participants which is funded by the drug company

Question: Semaglutide compared to placebo for weight loss

Certainty assessment							Impact	Certainty	Importance
№ of studies	Study design	Risk of bias	Inconsistency	Indirectness	Imprecision	Other considerations			
**Weight loss**									
8	randomised trials	serious[a]	not serious[b]	not serious	not serious	publication bias strongly suspected[c]		⊕⊕◯◯ Low	

CI: confidence interval

Explanations: a. The studies by Ahmann et al. (2018), Rubino et al. (2022), and Capehorn et al. (2020) are open-label studies, b. Only in the study by Rubino et al. (2022) was noticed intervention heterogeneity since the participants who were unable to tolerate 2.4 mg of semaglutide could receive 1.7 mg, c. The studies by Ahmann et al. (2018), Wadden et al. (2021), Rubino et al. (2022), Capehorn et al. (2020), Rubino et al. (2021), and Zinman et al. (2019) are small trials with 813, 611, 338, 577, 902 and 302 participants, respectively, which are funded by the drug companies

Question: Exenatide compared to placebo or dapagliflozin for weight loss

Certainty assessment							Impact	Certainty	Importance
№ of studies	Study design	Risk of bias	Inconsistency	Indirectness	Imprecision	Other considerations			
**Weight loss**									
3	randomised trials	not serious	not serious	not serious	serious[a]	publication bias strongly suspected[b]		⊕⊕◯◯ Low	

CI: confidence interval

Explanations: a. The study by Rodgers et al. (2021) do not provide sufficient information about OIS, b. All studies, Guja et al. (2018), Jabbour et al. (2018), and Rodgers et al. (2021) are small trials with 464, 695, and 182 participants, respectively, which are funded by the drug companies
